# Supplementary material for: Ferroptosis Inducer Improves the Efficacy of Oncolytic Virus-Mediated Cancer Immunotherapy
Source: Biomedicines. 2022 Jun 15;10(6):1425. doi: 10.3390/biomedicines10061425 (PMC9219720; doi:10.3390/biomedicines10061425)
Supplement: Supplementary file 1 [file biomedicines-10-01425-s001.zip › biomedicines-1668652-supplementary.pdf]

Supplementary Figures

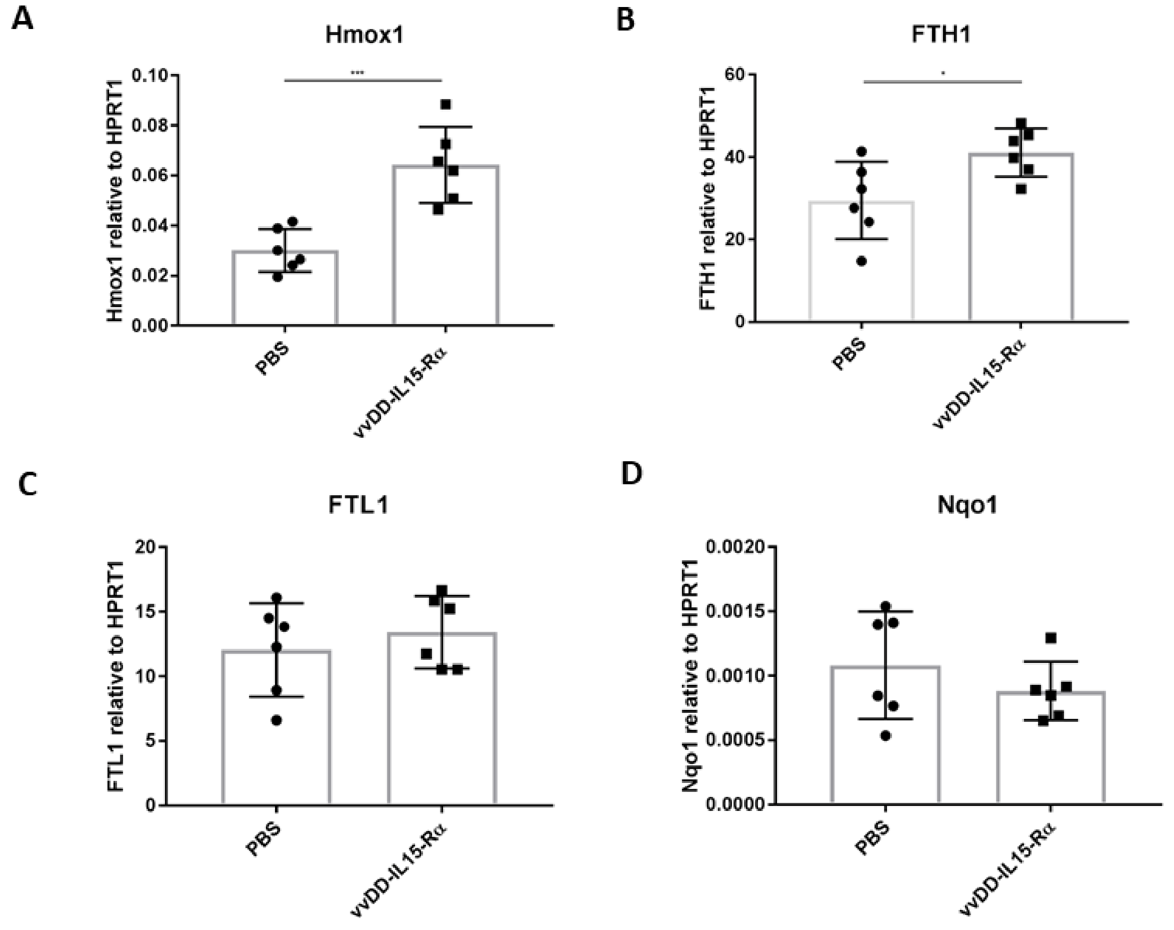

Figure S1. Change of Ferroptosis markers in MC38 colon cancer cells infected by the OV vvDD-IL15-Rα. The experimental conditions are as described in Figure 2. The relative levels of mRNA expression of these marker genes were determined by RT-qPCR compared to that of housekeeping gene HPRT1. A). Hmox1. B). FTH1. C).FTL1. and D). Nqo1. * $p < 0.05$; *** $p < 0.001$.

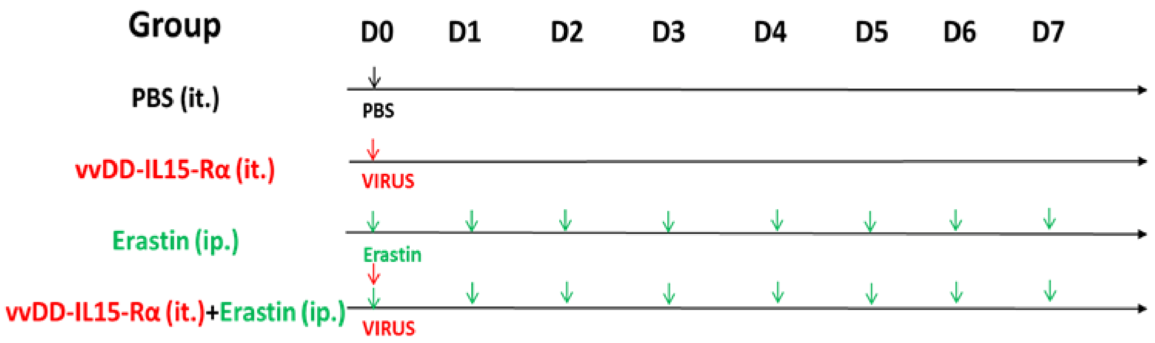

Figure S2. Time schedule of administration of different regimens. C57BL/6 (B6) mice were inoculated s. c. with 5.0e5 MC38-luc or 2.0e6 Hepa 1-6 cancer cells. They were randomly split into groups and treated with vvDD-IL15-Rα or/and Erastin after tumor volume reached ~5 × 5 mm2 as time schedule indicated. vvDD-IL15-Rα were intratumorally injected (i.t.) with 2.0e7 pfu/50 µL for only one injection, Erastin were intraperitoneal injection (i.p) daily with 20 mg/kg for total 7 injections.

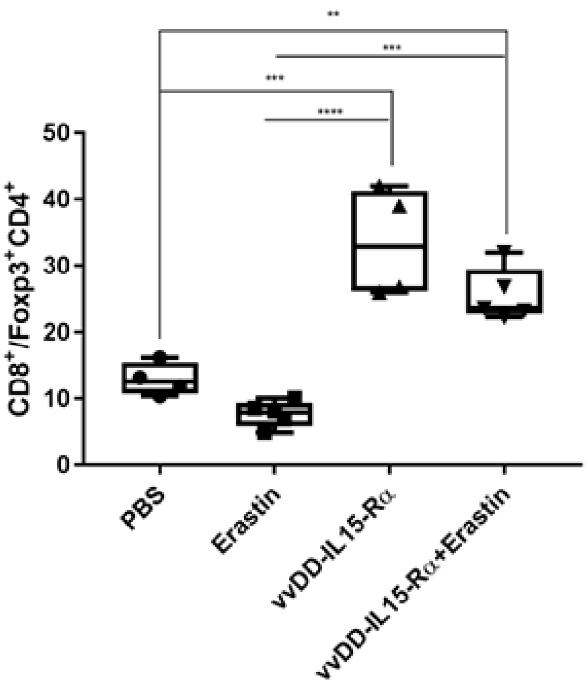

Figure S3. Combination therapy increase the ratio of CD8+/Treg cells in spleen. B6 mice were implanted s. c. with MC38-luc tumor cells and treated. Mice were sacrificed on 8 days after first treatment. The spleens were harvested for flow cytometry. The ratio of CD8+/Treg cells in four groups are shown. The statistical symbols are, **, $p < 0.01$; ***, $p < 0.001$; ****, $p < 0.0001$.
